# The Pathologic Roles and Therapeutic Implications of Ghrelin/GHSR System in Mental Disorders

**DOI:** 10.1155/2024/5537319

**Published:** 2024-11-26

**Authors:** Qianshuo Mao, Jinjia Wang, Zihan Yang, Ruidong Ding, Shuangyu Lv, Xinying Ji

**Affiliations:** ^1^Henan International Joint Laboratory for Nuclear Protein Regulation, School of Basic Medical Sciences, Henan University, Kaifeng 475004, Henan, China; ^2^Department of Neurosurgery, The First Affiliated Hospital of Henan University, Henan University, Kaifeng 475001, Henan, China; ^3^Faculty of Basic Medical Subjects, Shu-Qing Medical College of Zhengzhou, 6 Gong-Ming Road, Mazhai Town, Erqi District, Zhengzhou 450064, Henan, China; ^4^Department of Medicine, Huaxian County People's Hospital, Huaxian 456400, Henan, China

**Keywords:** anorexia nervosa, anxiety, bulimia nervosa, depression, ghrelin

## Abstract

Ghrelin is a hormone consisting of 28 amino acids. Growth hormone secretagogue receptor (GHSR) is a receptor for ghrelin, which is expressed in the brain, pituitary gland, and adrenal glands, especially in the hypothalamus. The binding of ghrelin to the receptor 1a subtype mediates most of the biological effects of ghrelin. Ghrelin has a close relationship with the onset of psychosis. Ghrelin can affect the onset of psychosis by regulating neurotransmitters such as dopamine, γ-aminobutyric acid (GABA), and 5-hydroxytryptamine (5-HT) through the hypothalamus–pituitary–adrenal (HPA) axis, brain–gut axis, the mesolimbic dopamine system, and other ways. Ghrelin activates neuropeptide Y (NPY) in the hypothalamic arcuate nucleus (ARC) through the GHSR. Ghrelin binds to neurons in the ventral tegmental area (VTA), where it promotes the activity of dopamine neurons in the nucleus accumbens (NAcs) in a GHSR–dependent way, increasing dopamine levels and the reward system. This article summarized the recent research progress of ghrelin in depression, anxiety, schizophrenia, anorexia nervosa (AN), and bulimia nervosa (BN), and emphasized its potential application for psychiatric disorders treatment.

## 1. Introduction

Ghrelin is an acylated peptide hormone of 28 amino acids, which synthesized by endocrine cells of the gastric mucosa. It was discovered by Masayasu Kojima, Kenji Kangawa, and colleagues in 1999 [1, 2]. It activates hypothalamic orexigenic neurons to increase food intake and body weight gain which promotes obesity [3]. Ghrelin receptors are distributed in the central nervous system (CNS) regions which have a crucial influence on depressive behaviors, such as amygdala, hippocampus, nucleus accumbens (NAcs), and ventral tegmental area (VTA) [4]. Ghrelin binds to its receptor and can affect multiple neurotransmitter systems, including dopamine, norepinephrine, and serotonin, which play crucial roles in the occurrence and development of mental disorders [5]. Consequently, ghrelin may influence the occurrence and development of mental disorders by regulating the function of these neurotransmitter systems. Research suggests a strong link between ghrelin and mental disorders [6, 7]. This article aims to give a summary of the most recent progress for comprehending the signaling pathways and the physiological and pathophysiological functions of ghrelin in mental health disorders such as depression, anxiety, schizophrenia, anorexia nervosa (AN), and bulimia nervosa (BN).

## 2. Ghrelin and Growth Hormone Secretagogue Receptor (GHSR) System

In human genetics, the coding gene for the hunger hormone ghrelin is known as ghrelin and obestatin prepropeptide *(GHRL)*. It is located in the 3p25 region of chromosome 3 [1]. The *GHRL* gene consists of four exons and three introns and encodes a preproghrelin protein. The ghrelin gene is expressed in various tissues and organs throughout the human body and is widely distributed throughout the genome. Apart from gastric cells, low levels of expression are found in other tissues including the brain, pancreas, and intestines [1]. The preproghrelin protein is processed by ghrelin O-acyltransferase (GOAT) into ghrelin and binds to ghrelin receptors to regulate appetite [8]. There is another subtype of des-acyl ghrelin in the circulatory system, and their functions are different [9]. Multiple studies have suggested that des-acyl ghrelin may exert its function by binding to receptors other than ghrelin receptors [10]. Des-acyl ghrelin can counteract the increased appetite caused by peripheral application of ghrelin [11]. In addition, in terms of neurogenesis, des-acyl ghrelin and acyl-ghrelin may play opposite roles, and changes in their ratios may be associated with certain neurodegenerative diseases [12, 13]. GOAT, as an enzyme capable of acylating ghrelin, is highly expressed in the stomach and intestines, suggesting its potential target role in regulating appetite and suppressing obesity [8]. The expression of GOAT in the hypothalamus and pituitary gland [14] further suggests that GOAT may exert its function by regulating the ratio of acylated and des-acyl-ghrelin.

On the other hand, the *GHSR* gene is a G-protein-coupled receptor located on chromosome 3 at the 3q26.31 region. It encodes the GHSR protein [15]. The predominant expression of GHSR1a can be observed in various organs including the anterior pituitary gland, pancreatic islets, adrenal gland, thyroid, myocardium, arcuate nucleus (ARC), hippocampus, substantia nigra pars compacta (SNpc), VTA, and raphe nuclei [16]. But the hypophysis is the primary site of expression for both its receptor GHSR and its variants, and other tissues have far lower expression levels than it does [17]. In the stomach, GHSR is mainly concentrated in epithelial cells and neurons [18]. In the intestines, it is predominantly found in the small and large intestines, where it regulates intestinal motility and gastrointestinal secretion, thereby impacting the speed and absorption of food through the intestines [19]. GHSR is also widely distributed in adipose tissue, especially white adipose tissue, and influences the growth and metabolism of adipose tissue [17].

**Figure 1:**
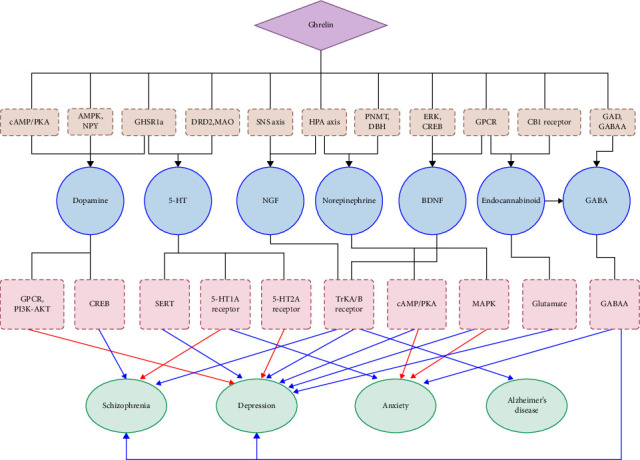
The mechanism and effect of ghrelin on anxiety, depression, and schizophrenia. 5-HT, 5-hydroxytryptamine; AMPK, AMP-activated protein kinase; cAMP, cyclic adenosine monophosphate; CB1, type-1 cannabinoid receptor; CREB, cAMP response element-binding protein; DBH, dopamine beta-hydroxylase; DRD2, dopamine receptor D2; ERK, extracellular signal-related kinase; GABAA, *γ*-aminobutyric acid A; GAD, glutamic acid decarboxylase; GHSR1a, growth hormone secretagogue receptor 1a; GPCR, G protein-coupled receptor; HPA axis, hypothalamic–pituitary–adrenal axis; MAO, monoamine oxidase; MAPK, mitogen-activated protein kinase; NPY, Neuropeptide Y; PKA, protein kinase A; PNMT, phenylethanolamine N-methyltransferase; SERT, 5-HT transporter; SNS, sympathetic nervous system. Image created with figdraw.com.

In addition, ghrelin can regulate depression by affecting the expression of neurotrophic factors. Neurotrophic factors are a type of protein that contributes to neuronal growth and survival, including nerve growth factor (NGF) and brain-derived neurotrophic factor (BDNF). Ghrelin affects the NGF signaling pathway by activating the HPA axis and the sympathetic nervous system (SNS) [20]. Through the activation of the tropomyosin receptor kinase A (TrkA) receptor and associated signaling pathways, NGF plays a crucial role in neuronal survival, synaptic plasticity, and neurotransmitter release [21]. NGF also affects the occurrence and development of mental disorders by regulating inflammatory responses, oxidative stress, and neurotransmitter release [20, 22]. Inflammatory responses also play a role in depression, and NGF can alleviate inflammatory responses, which may have an effect on depression treatment [20, 23]. Ghrelin promotes the expression and release of BDNF by activating G protein-coupled receptors (GPCRs) and the extracellular signal-regulated kinase (ERK) signaling pathway that depends on G proteins [24, 25]. Ghrelin can promote the transcription and expression of the BDNF gene by activating the cyclic adenosine monophosphate (cAMP) response element-binding protein (CREB) signaling pathway [26]. Activating its receptor TrkB, BDNF promotes neuronal survival and neurotransmitter release. TrkB plays a crucial role in depression, epilepsy, and Alzheimer's disease [27–29]. Ghrelin affects the occurrence and development of other mental disorders, as shown in Figure 1.

## 3. Depression

**Figure 2:**
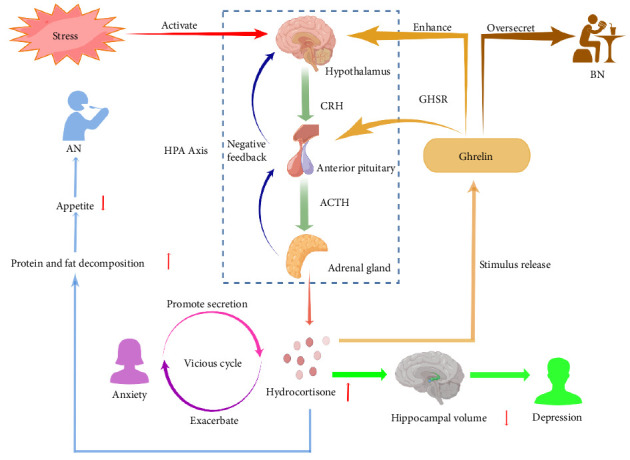
The effect of Ghrelin on depression, anxiety, AN, and BN via the HPA axis. When the body is stressed, the hypothalamus releases CRH, which then stimulates the pituitary gland to release ACTH. This triggers the adrenal glands to release cortisol. The feedback is negative. Elevated cortisol levels can stimulate the secretion of ghrelin. They form a positive feedback. ACTH, adrenocorticotropic hormone; AN, anorexia nervosa; BN, bulimia nervosa; CRH, corticotropin-releasing hormone; GHSR, growth hormone secretagogue receptor; HPA axis, hypothalamus–pituitary–adrenal axis. Image created with figdraw.com.

Ghrelin affects depression via hypothalamus–pituitary–adrenal (HPA) axis functions. The HPA axis is dysregulated in persons with severe mental illness, including depressive symptoms, with either low or high activity [30, 31]. Ghrelin likely plays a role in regulating the body's response to stress and changes in nutrition and metabolism. It does this by releasing adrenocorticotropic hormone (ACTH) and cortisol in humans, which helps regulate mental disorders like depression, anxiety, AN, and BN [32].  Figure 2 demonstrates the effect of ghrelin on depression, anxiety, AN, and BN via the HPA axis. In the early clinical data analysis, it was shown that there was a negative correlation between depression and hippocampal volume, excluding the interference of age and gender. After drug treatment, with the decrease in cortisol level, the hippocampal volume of patients tends to increase [33]. However, according to several studies, it has been demonstrated that injection of ghrelin can increase the release of short-term endothelin and GH, which can have therapeutic effects on depression [34]. Moreover, a study has revealed that ghrelin can alleviate anxiety following short-term stress by activating the HPA axis [35]. In addition to being affected by hormones in the body, patients with depression also exhibit specific changes in their brain waves during sleep [36], and they may be treated by adjusting changes in brain waves. Research shows that ghrelin injection can increase non-rapid eye movement sleep and inhibit rapid eye movement sleep in human subjects [34, 37]. It has a therapeutic effect on depression, but there are gender differences in this effect. It was indicated that GOAT inhibitors could prevent the rat HPA axis from functioning, suggesting a potential effect on the treatment of depression [38]. In addition, studies have established a significant correlation between the presence of *Helicobacter pylori* and the onset of depression, but the reason is unknown [39]. It may be that *H. pylori* can affect the expression of GOAT [40], or decrease the circulation level of ghrelin [41], thus inducing depression and anxiety.

Ghrelin participates in the communication that takes place in the gut and brain, and then affects depression [42]. Depression is commonly observed among individuals suffering from inflammatory bowel disease, with a higher occurrence of depression compared to the general population [43, 44]. The confluence of brain and intestinal metabolic alterations suggests that the brain-intestinal axis may be involved in depression in unexpected chronic mild stress-induced rat model of depression [45]. Previous studies suggest that ghrelin can stimulate gastric ulcer healing through the release of endogenous nitric oxide (NO), as well as vagus nerve and sensory nerve stimulation, and its mechanism may be that ghrelin can stimulate endogenous ghrelin and insulin-like growth factor-1 [46, 47]. The above research indicates that the gut microbiota regulates changes in gastrointestinal hormones, which in turn have an impact on the brain. It is suggested that in the future treatment of depression, more attention needs to be given to the role of ghrelin on gut microbiota.

Ghrelin can affect the level of dopamine, and the decline of dopamine levels is closely associated with depression. Dopamine is a type of neurotransmitter that has a significant impact on our reward and motivation systems. When we experience pleasant experiences or achieve goals, dopamine levels increase, producing a sense of satisfaction and happiness [48, 49]. In addition, dopamine is also associated with emotions. It participates in the regulation of positive emotions such as pleasure, happiness, and satisfaction, as well as negative emotions such as anxiety and depression. The imbalance of dopamine may lead to emotional disorders, such as depression bipolar disorder [50]. Dopamine, when combined with D1 and D2 receptors through the phosphatidylinositol 3-kinase (PI3K)/protein kinase B (PKB/AKT) signaling pathway and CREB, significantly influences neuronal development, synaptic plasticity, and the prevalence and management of mental illnesses [51–53]. A study has reported that the decrease in dopamine levels may trigger symptoms related to depression [54]. The mesolimbic dopamine system is formed by the NAc and VTA. In the VTA-NAc circuit of rodents, the manipulations of key proteins may produce behavioral phenotypes directly related to depression [55]. In addition, basic and clinical studies have shown the dopamine system is defective in patients with depression, and dopamine system defects contribute to the symptoms of pleasure deficiency in depression patients [54, 56]. A decrease in dopamine levels may diminish the functional connections in the brain's reward circuit, resulting in abnormal processing of reward and punishment behaviors in the brain. It weakens the individual's response to reward stimulation, thereby reducing positive emotions and happiness, promoting the occurrence of depression, or promoting symptoms of lack of pleasure and sustained tension in patients with depression [57–59]. These studies indicate a clear correlation between dopamine and depression. It was demonstrated that ghrelin stimulated the release of dopamine by activating VTA dopamine neurons, which in turn enhances appetite [60, 61]. When ghrelin was injected into the brain of mice, it combined with the neurons of VTA in mice and rats. It alleviated the mice's depressive-like behavior by increasing dopamine levels and promoting an increase in dopaminergic neural responses, synapse formation, and dopaminergic cycling in the NAc in a GHSR-dependent way [53, 62]. It has been found that systemic administration of the ghrelin receptor antagonist JMV2959 inhibited the ghrelin-induced release of dopamine from the NAc shell [63]. This suggests that ghrelin may alleviate or inhibit the occurrence and progress of depression by promoting secretion and release of dopamine.

Regulating the expression level of ghrelin is a promising treatment strategy for depression. Previous studies have shown that exogenous ghrelin has the ability to alleviate depression-like behavior in rodent models of myocardial infarction [64], high-fat diet, and circadian rhythm disorders [65]. Ghrelin not only showed the potential to alleviate depressive behavior in experimental mouse models, but the changes in its expression level can also serve as a potential indicator for evaluating the therapeutic effect of existing antidepressant drugs [66].

The high similarity in symptoms between depression and bipolar disorder complicates their clinical differentiation. Accelerating the speed and accuracy of this distinction is still an area that needs to be explored. Measuring ghrelin expression levels could serve as a potential tool for differential diagnosis. Studies indicate that ghrelin levels are higher in bipolar disorders compared to those with major depressive disorder (MDD) [67], yet remain lower than in healthy individuals. It was demonstrated that the hormone levels during the recovery phase following manic episodes in bipolar disorder revealed a decrease in serum acylated and total ghrelin levels, with elevated acylated ghrelin (AG) levels correlating with an increased number of manic episodes [68].

Variations in ghrelin expression levels may influence cognitive functions in bipolar disorders, with a positive correlation observed between executive functions and ghrelin levels [69]. The rising obesity rates among bipolar disorders adversely affect their prognosis and lifespan, potentially linked to alterations in metabolic functions within these patients [69]. Bipolar disorders individuals exhibit greater food cravings compared to the general population, with a notable negative correlation observed between food cravings and ghrelin levels [70]. Compared to controls, bipolar disorders showed lower serum glucagon and glucagon-like peptide (GLP-1) levels and higher gastric inhibitory polypeptide (GIP) levels, suggesting alterations in glucose metabolism, which may contribute to the elevated obesity rates in bipolar disorders [71].

## 4. Anxiety Disorder

Patients with anxiety disorder often exhibit symptoms of reduced appetite and eating disorders, which may be related to insufficient ghrelin secretion [72]. Some studies suggest that patients with anxiety disorder may inhibit the secretion of ghrelin due to emotional instability and tension, leading to reduced appetite, while exogenous ghrelin can alleviate symptoms of anxiety disorder [6, 73]. AG, unacylated ghrelin (UG), and copeptin levels rose in a study including individuals who had attempted suicide when their anxiety levels increased [74]. The decrease in ghrelin levels may be one of the reasons why patients with anxiety disorder experience nausea and vomiting [75]. The people with anxiety disorders had greater ghrelin levels than people in the general population [76]. These findings indicate a possible relationship between ghrelin and the occurrence and severity of anxiety disorder.

Ghrelin regulates stress and emotional responses by interacting with the HPA axis. Research has revealed that ghrelin can stimulate the activation of the HPA axis, leading to increased cortisol levels and modulation of stress responses, thereby affecting the occurrence and severity of anxiety disorders [77, 78]. In addition, ghrelin can regulate neurotransmitters in the brain that are closely related to emotional regulation, such as dopamine and norepinephrine. Ghrelin can influence the emotional state of patients with anxiety disorders by affecting the levels of the neurotransmitters [6, 25]. In terms of neural regulation, ghrelin can inhibit the activity of hippocampal neurons by interacting with the hippocampus–hypothalamus–amygdala (HHA) neural circuit, thereby increasing the incidence of anxiety disorders [6].

Ghrelin can also regulate emotional and cognitive functions by interacting with the neuro transmitters glutamate and *γ*-aminobutyric acid (GABA) [7]. It was demonstrated that ghrelin can decrease GABA release and block GABA neuron activation, thereby exacerbating the occurrence of anxiety disorders and the symptoms in patients with anxiety disorders [7, 79]. Abnormal expression of the 5-hydroxytryptamine (5-HT)1A receptor may be related to the development of anxiety symptoms in patients with anxiety disorders [80, 81]. Ghrelin can directly increase the activity of GABAergic neurons and promote GABA release by activating glutamic acid decarboxylase (GAD) [82]. The sensitivity and functionality of GABAA receptors can also be controlled by ghrelin, which can also change the subunit ratios and increase the production of GABAA receptor subunits [83]. Mental illnesses can arise due to disturbed GABA signaling caused by aberrant expression of GABAA receptors and abnormal synthesis and release of GABA [84, 85].

Currently, the role of ghrelin based on animal model studies is emerged.  Table 1 lists a portion of the change of ghrelin in different mouse models and experimental conditions. In animal experiments, ghrelin plays a dual role in anxiety animal models. Administration with ghrelin, either centrally or peripherally, can reduce anxiety-like behaviors [35, 86, 90]. In the mouse model, ghrelin gene knockout mice show higher anxiety levels compared to wild-type mice after acute restraint, and exogenous ghrelin can alleviate this change [35]. By injecting exogenous growth hormone-releasing peptide or restricting calorie intake to increase growth hormone-releasing peptide levels, and in experiments involving forced swimming and an elevated plus maze, mice exhibit less anxiety-like behavior [6]. Furthermore, long-term peripheral administration of ghrelin can alleviate anxiety caused by intense stress [86]. When growth hormone-releasing peptide receptor knockout mice are subjected to acute calorie restriction, their anxiolytic and depressive effects are dependent on the interplay between endogenous growth hormone-releasing peptide and growth hormone-releasing peptide receptor [91, 92]. This shows that raising ghrelin levels can help treat anxiety-like behaviors induced by both short-term and long-term stress. Furthermore, ghrelin protects neurons by inducing hippocampus neural stem cell growth [90] or preventing the nuclear factor kappa B (NF-*κ*B) signaling pathway and the NLRP2 inflammasome from activating [93]. Conversely, several studies have showed a link between elevated ghrelin levels and behaviors exacerbating anxiety [94]. Injection with ghrelin into the cerebral ventricles of mice can induce anxiety behavior [7, 95]. Based on this, injection ghrelin within various hypothalamic regions in mice can reveal different sensitivities to anxiety behaviors [96], suggesting different roles of ghrelin in different regions in mouse mental activities. Injection with a ghrelin receptor (GHSR1a) inverse agonist PF-04628935 before injection ghrelin into the dorsal nucleus raphe in mice can alleviate ghrelin-induced anxiety behavior [97]. Based on this, the mechanism of ghrelin was investigated. The majority of studies indicate that ghrelin regulates anxiety via the HPA axis [35, 83, 98]. However, in recent years, more and more research has shown that ghrelin also acts through the central serotonin system [99]. Increasing ghrelin receptor signaling in the amygdala can produce anxiolytic effects and decrease the expression of serotonin receptors [100]. Moreover, administration with acute ghrelin enhances serotonin turnover in the amygdala and increases serotonin receptor expression in mice, which prevents ghrelin from inducing anxiety [101]. Theses indicate that ghrelin acts not just through the HPA axis but also through the central serotonin system.

In short, there is a complex relationship between ghrelin and anxiety disorders. In rodent models, ghrelin has been observed to alleviate anxiety-related behaviors induced by mild stress [86], but a detailed understanding of its underlying mechanisms is still an ongoing research field. Future research needs to further explore the relationship and find new treatment methods to help patients with anxiety disorder restore normal ghrelin levels.

## 5. Schizophrenia

Individuals with schizophrenia have altered the level of ghrelin compared to healthy individuals, and antipsychotic medications can also affect ghrelin levels. Schizophrenia is one of the most widespread mental illnesses, and individuals with schizophrenia have a higher prevalence of obesity than the general population [102]. This may be related to ghrelin, a hormone associated with hunger.

Ghrelin is associated with schizophrenia through its impact on brain inflammatory response. Numerous research findings indicate an increased occurrence of metabolic syndrome and diabetes among individuals with schizophrenia, who, in comparison to the broader population, exhibit higher ghrelin levels [103, 104]. However, some studies indicate a decrease in ghrelin levels in individuals with schizophrenia [105]. Dysregulated activation of proinflammatory cytokines and microglia cells is thought to be a potential cause of schizophrenia [106, 107]. While ghrelin, as a broad anti-inflammatory agent, can lessen the inflammatory factors' release [108], suppress the triggering of microglia cells [109, 110], and thus decrease the inflammatory response and alleviate oxidative stress [111, 112].

Ghrelin affects the levels of different neurotransmitters, thus contributing to the emergence and advancement of schizophrenia. Ghrelin may affect the onset of schizophrenia by influencing the dopamine neurotransmitter system. Presently, the dopamine hypothesis, which postulates that elevated dopamine neurotransmission may be the source of schizophrenia symptoms, is the most commonly recognized neurochemical explanation for the disorder [113]. It is thought that people with schizophrenia have higher dopamine levels in their brains than people without the disorder, which may explain symptoms like delusions and hallucinations [113–115]. Ghrelin may enhance the secretion of dopamine and affect the activity of dopamine receptor genes, consequently elevating dopamine concentrations [53, 116]. There is a negative feedback regulation between ghrelin and serotonin receptors [117], and clinical data show a significant decrease in the mRNA expression of GHSR1a in individuals with schizophrenia [118]. These findings suggest that ghrelin might play a role in the development of schizophrenia. Furthermore, the oxytocin receptor (OXTR) have been shown to interact with the ghrelin receptor (GHSR), suggesting that ghrelin-targeted therapy may be able to control oxytocin signaling associated with schizophrenia [119]. Oxytocin signaling is associated with various disorders, including obesity, autism, schizophrenia, and depression. It was reported that OXTR and GHSR can form heterocomplexes, leading to significant changes in downstream OXTR signaling, which may also be relevant to the mechanism through which ghrelin affects schizophrenia [120]. Additionally, ghrelin can affect neurons grow and mature. Aberrant neural connections, neuronal loss, and aberrant neuronal activity are all associated with the emergence of negative symptoms linked to schizophrenia [121–123].

Medication used to treat schizophrenia may impact ghrelin levels. This suggests a significant association between ghrelin and schizophrenia. Many studies have demonstrated that people with schizophrenia who take second-generation antipsychotic drugs like olanzapine and risperidone frequently develop symptoms like diabetes and obesity, which may be because that the second-generation antipsychotics might increase the production of ghrelin [103]. Olanzapine is a preferred medication for treating schizophrenia and is known to contribute to weight gain [124]. Both the plasma ghrelin content and the positive symptoms on the positive and negative symptoms scale decreased following a 16-week course of olanzapine medication [125]. The antagonist effect of olanzapine on muscarinic M3 receptors in the brain can regulate the release of growth hormone-releasing peptides by inhibiting the vagus nerve [126].

The influence of antipsychotic drugs on ghrelin levels is different. Studies demonstrated that treatment with atypical antipsychotic (AAP) drugs increased ghrelin levels [127, 128], while ghrelin levels did not change or decrease following AAP treatment, according to previous research [129–131]. There is also research suggesting that long-term use of antipsychotic drugs may disrupt the negative feedback mechanism of ghrelin [132]. Medication intake may result in reduced ghrelin levels within the body [130, 133], leading to an imbalance between ghrelin and leptin [134], as well as changes in the ratio of acylated and unacylated growth hormone-releasing peptides [135], resulting in obesity.

## 6. AN

AN is a severe and persistent eating disorder. Patients suffer from psychogenic loss of appetite and extreme, life-threatening wasting. The mortality rate is on the rise annually as society progresses [100]. Up to 20% of patients develop persistent or even lifelong AN [136]. The disease is associated with disorders of the endocrine system, psychosocial factors, and genetic factors. Hormone therapy is currently an area of investigation. Presently, most commonly prescribed medications primarily target the CNS.

The primary clinical indication in individuals afflicted with AN is an absence of appetite or a decline in appetite. This may be related to the disruption of the balance between ghrelin and leptin in the patient's body. Ghrelin acts as an appetite-regulating hormone by binding to CNS ghrelin receptors, inducing food intake, and antagonizing the effects of adipocyte-derived leptin in vivo. Several clinical studies have found that circulation levels of growth hormone (GH) are significantly elevated among patients [137–139], while leptin levels are decreased [140]. Ghrelin levels decreased as patients gained weight during treatment [137, 141]. The theory suggests that increased ghrelin levels could stem from insufficient adjustment to energy consumption. A line of research indicates that the ghrelin levels were changed after a diet, and by comparing ghrelin levels after meals, and it is evident that food has a diminished capacity to inhibit ghrelin secretion [142–144]. Different diets have different effects on the release of ghrelin, and the strength of the inhibitory effect of high-carbohydrate and high-protein breakfasts on the release of ghrelin and the time required to reach the low level of ghrelin are different [145], indicating that the level of ghrelin in patients suffering from AN is regulated in response to the variance in energy.

The expression level and number of receptors of ghrelin affect AN. The contrast between high levels of ghrelin and low appetite suggests that the AN may be less sensitive and more resistant to ghrelin. In several subsequent experiments in which subjects were injected with exogenous ghrelin, it was observed that patients with AN exhibited reduced sensitivity to ghrelin concerning the release of growth hormones and appetite [138, 140]. Certain investigations have revealed that those suffering from AN also have a lower number of ghrelin receptors, which may further affect the action of the ghrelin hormone [146]. In recent studies, mRNA levels of *GHSR1a* in the hypothalamus of mice suffering from AN have been found to be decreased [147], and increased methylation of the GHSR1a promoter is observed in short-weighted patients with AN [148]. These suggest that lower number of ghrelin receptors could be a factor in regulating appetite in people with AN. Concurrently, this discovery reveals the probable epigenetic regulatory pathways of the ghrelin signaling system, especially when ghrelin levels are high. The immunoglobulin (Ig) G autoantibodies to ghrelin in patients with AN primarily exist as immune complexes with deacylated gastric emptying hormone, resulting in a reduction of the unbound amount of ghrelin [149], reflecting adaptive changes in the patient. Genetic elements are vital in the development of AN, with genome-wide association studies confirming link between ghrelin gene polymorphisms and the prevalence of AN, suggesting the presence of a ghrelin-associated genetic component in patients with AN [150]. Between 5% and 10% of patients with AN are female relatives who also suffer from eating disorder [151]. Studies have shown that genetic variation in GOAT is linked to AN [152].

Food-related motivation is also altered to some extent in people with AN. Ghrelin can affect AN by influencing the food reward system. By measuring changes in peripheral ghrelin levels, a disturbance in growth hormone-releasing peptide regulation of food-related feelings of pleasure and reward can be found in patients with AN [153]. Ghrelin affects dopaminergic neuron activity, which modulates appetite and reward behavior. In patients with AN, dopaminergic neuron activity is abnormal, leading to decreased appetite and feelings of food reward, which is further exacerbated by the lack or suppression of ghrelin, thus exacerbating the symptoms of AN [154]. Studies indicate that individuals with AN exhibit reduced dopamine and 5-HT levels and higher levels of GABA in certain regions of the brain, which contribute to decreased appetite and emotional instability [155, 156]. In addition, ghrelin induces changes in dopamine and NAc cell activity in the NAc in vivo mouse model [157]. These studies reflect the regulative function of ghrelin in the food-related reward system [158], and direct evidence and continued exploration are needed.

**Figure 3:**
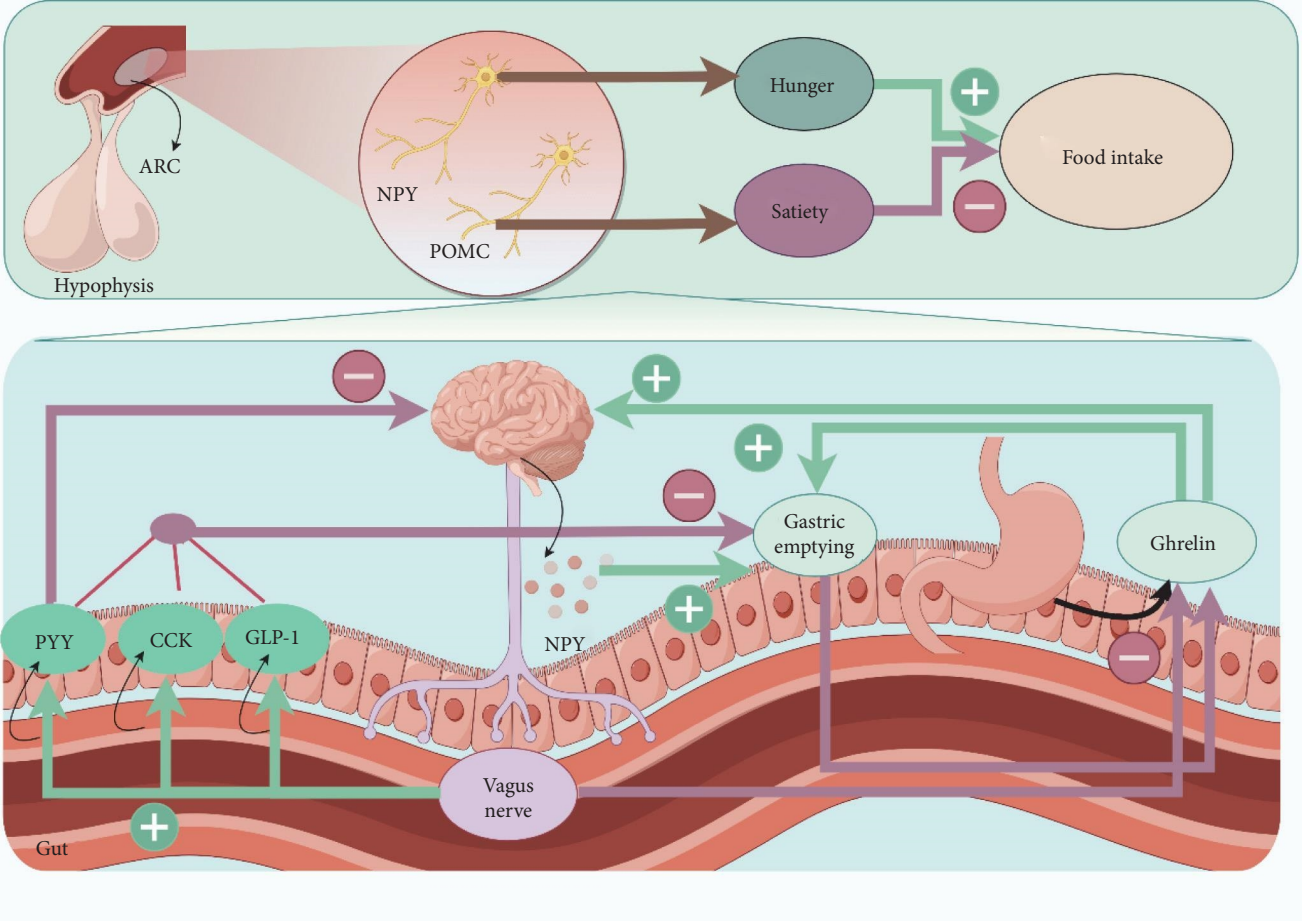
Ghrelin and other hormones affect AN via gastric emptying and food intake. The vagus nerve is the major determinant of the inhibition of ghrelin secretion and gastric emptying (purple arrow, −) and the stimulation of CCK, GLP-1, and PYY secretion (green arrow, +). PYY is an appetite-suppressing peptide, while NPY is an appetite-promoting peptide. PYY has an inhibition function to PYY. NPY stimulates gastric emptying (green arrow, +), while CCK, GLP-1, and PYY inhibits gastric emptying (purple arrow, −). ARC, arcuate nucleus; CCK, cholecystokinin; GLP-1, glucagon-like peptide; NPY, neuropeptide Y; POMC, pro-opiomelanocortin; PYY, peptide YY. Purple arrow: inhibition and green arrow: promotion. Image created with figdraw.com.

Food restriction-induced hypermobility is partially present in patients with AN, and this may be related to activation of the ghrelin signaling system in the CNS. Research has shown that intraperitoneal injection of ghrelin into mice stimulates the limbic dopamine system in the midbrain and induces an increase in activity in mice [159]. On this basis, it was empirically noted that the introduction of a ghrelin receptor activator resulted in an augmentation of voluntary locomotion in *ghrelin* knockout mice [60]. In contrast to ghrelin antagonists, which reduced food anticipation in mice, antagonist administration had no impact on food consumption in activity-based anorexia mouse model [160]. In addition, the deacylated ghrelin has been correlated with food restriction and voluntary exercise [161]. The evidence presented above implies that a decrease in energy consumption may result in a heightened activation of the ghrelin signaling system, which in turn amplifies the body's inclination for voluntary movement, implying that ghrelin receptor antagonists may have a therapeutic effect. Furthermore, other studies showed that ghrelin levels in patients with AN are comparable to those without the disorder. In 2001, it was first shown that fasting plasma grades of ghrelin were elevated in AN [137]. The patients with AN exhibit reduced ghrelin responsiveness, causing hunger insensitivity even during starvation [162]. In addition, previous study indicates that ghrelin in the blood of patients with AN is similar to those in healthy people before and after eating, but the levels are significantly lower during eating. This can be associated with the emotional response and cognitive processing of food in patients with AN [163, 164]. However, it was demonstrated notable dissimilarities between the fasting plasma ghrelin grades of AN patients and those of age-equal healthy controls, in addition to elevated ghrelin levels in AN patients [137]. There is an increase in both central and peripheral ghrelin expression, but postprandial ghrelin signaling is hindered due to a delay or cessation of postprandial ghrelin decline [144, 165]. Patients with AN have facilitated HPA axis activity, leading to elevated cortisol levels in the body, which affects appetite and metabolism [162, 166]. Neuropeptide Y (NPY) is an appetite-promoting peptide while peptide (PYY) is an appetite-suppressing peptide, and patients with AN have reduced standards of NPY and increased standards of PYY, which may lead to disturbances in appetite control [167, 168]. AN has a connection to gastric emptying, which reduces the rate of gastric emptying. Inhibition of gastric emptying may worsen appetite suppression in AN by increasing feelings of satiety [169]. After blocking the vagus nerve, the secretion of ghrelin is increased, while the secretion of cholecystokinin (CCK) and GLP-1 is decreased [170, 171]. The CCK, GLP-1, PYY, NPY, and ghrelin affect AN via gastric emptying and food intake (Figure 3).

In addition to its possible association with the development of AN, exogenous ghrelin may also be used to treat AN. Intraperitoneal injection of ghrelin reduced the reduction in food intake and inhibited the progression of active AN in mice, but the mice still lost weight [172]. Exogenous ghrelin can bind to ghrelin antibodies in the plasma of obese individuals, thereby protecting endogenous ghrelin from degradation, and thus enhancing the pro-appetite effects of ghrelin [173]. Chronic intravenous administration of ghrelin (twice daily for 14 days) at a dose of 3 μg/kg body weight has been shown to increase daily energy intake and improve gastric discomfort and constipation in patients with AN, which may be related to increased gastric motility [174, 175]. Studies have demonstrated that growth hormone can be advantageous in the management of AN, and the growth hormone enhancer rikkunshito has also been demonstrated to be involved in the management of AN. In human trials, the rikkunshito relieved nausea and vomiting, caused an increase in appetite in chemotherapy-induced AN, and may also cause an increase in ghrelin levels, which could be used to address ghrelin resistance [176, 177].

The correlation between ghrelin and AN continues to be a subject of debate, although patients with AN generally exhibit reduced levels of ghrelin. Studies indicate a decrease in ghrelin production in AN patients, with those suffering from AN showing a reduction exceeding 30% in ghrelin levels relative to healthy individuals. The reason could be the patients' dietary restrictions and weight control behaviors. Ghrelin injections can improve appetite and increase food intake in patients with AN [160, 178].

## 7. BN

The ghrelin level may be related to the onset of BN in patients. Ghrelin activates NPY in the ARC of the hypothalamus via GHSR, thereby, increasing appetite and promoting weight gain [179, 180]. BN is an appetite disorder in which patients exhibit uncontrollable overeating and binge eating behaviors. Clinical experiments demonstrated that basal ghrelin levels present in BN subjects were markedly elevated compared to those in healthy subjects., and patients with BN have higher levels of postprandial ghrelin [181, 182]. This indicates that BN patients have an increased sensitivity to ghrelin, which results in the production of more ghrelin and subsequently leads to increased appetite and disrupted eating control [183, 184]. Research indicates that in patients with bulimia, the descending reaction of circulating growth hormone–releasing peptides is diminished following food consumption, possibly due to a blunted growth hormone–releasing peptide response to food intake in BN, which in turn causes impaired inhibition of feeding drive [146, 182].

The interaction of ghrelin with the nervous system may also be involved in the development of BN. Studies have shown that ghrelin can regulate appetite and satiety by interacting with neurons in the hypothalamus and brainstem, affecting the release and synthesis of a variety of neurotransmitters and neuropeptides [185]. In BN subjects, the rise in PYY levels after meals is also blunted [182]. In addition, ghrelin has the capacity to alter the functioning of brain areas linked to emotional control, thus altering how mood and stress impact hunger [186]. These findings provide an important reference for further research on the mechanism and treatment of BN.

Ghrelin is associated with mood and reward systems, which may also play a role in the development of BN. Studies have shown that ghrelin plays a role in controlling hunger and eating habits by altering the secretion and production of neurotransmitters like dopamine, which in turn affects the reward mechanism and the sensory perception of food [187]. Dopaminergic function in the brain is important in the control of emotions and behaviors associated with the brain's reward system [61]. When ghrelin attaches to the cells in the SNpc region, it triggers the electrical activation of dopamine neurons in the same region. This ultimately results in augmented concentrations of tyrosine hydroxylase messenger ribonucleic acid (mRNA) and dopamine within the dorsal striatum [188]. Meanwhile, ghrelin enhances the preference for high-fat and high-sugar foods, both possibly contributing to the onset and development of BN [186, 189]. In addition, ghrelin can influence the activity of the functioning of brain areas linked to emotion regulation, thereby modulating the effects of mood and stress on appetite [190]. It has been demonstrated that ghrelin maintains a prolonged suppressive impact on inflammation caused by lipopolysaccharides, potentially linked to the excessive response to food incentives in bulimia sufferers.

Therefore, there is a relationship between ghrelin and BN. However, as research in this area is still ongoing, the precise mechanism between them is still unclear, and further studies will help us to better understand this relationship and provide new ideas for the prevention and treatment of BN.

## 8. Conclusion

Ghrelin not only serves as a messenger, relaying the peripheral nutritional status to the brain to uphold the body's energy balance, but it also plays a pivotal role in modulating the CNS's functionality through diverse mechanisms. Ghrelin can alter the mood state and cognitive function of patients by affecting the activity of the dopamine system and reward mechanism, thus impacting the development of mental disorders. Ghrelin has the ability to alter stress and emotional reactions by affecting the HPA axis's function, potentially playing a role in the emergence of anxiety disorders, posttraumatic stress disorder, and similar mental health conditions. At present, some studies have achieved certain therapeutic effects by injecting exogenous ghrelin into mice for the treatment of mental disorders. Additionally, certain investigations have identified alterations in ghrelin expression levels as an indicator for differential diagnosis of diseases or the progression of disease courses.

These findings suggest that ghrelin may serve as a novel target for the treatment of mental disorders. Hopefully, more effective and safe ghrelin-based medications can be developed and applied in the treatment of mental disorders. Nonetheless, additional studies are required to investigate ghrelin's mechanisms, safety, specificity, and sustained effectiveness in treating mental illnesses. Techniques such as functional magnetic resonance imaging and electroencephalography can be utilized to reveal its regulatory effects on neural electrical activity, functional connectivity, and brain region activity, thereby further investigating the neural mechanisms related to mental disorders. More clinical experimental studies using randomized controlled trials with double-blind designs can be employed to validate the therapeutic effects of ghrelin.

## Figures and Tables

**Table 1:** The change of ghrelin in different mouse models and experimental conditions.

Experimental study	Experimental subjects	Experimental processing	Experimental result	References
Anxiety/depression	Male C57BL/J6 and male SD rats	CUMS	↑AG	[86]
	60% caloric restriction mice for 10 days	EPM and FST	↑AG	[6]
	Wild-type C57BL6/J mice	10 days of CSDS	↑AG	[6]
	Wild-type mice	10 days of CSDS	↑AG	[77]
	WKY female rats	WAS	↑AG	[87]

Eating disorder	Male C57Bl6 mice	12 weeks on HFD	↓TG, ↓AG	[88]
	Diet-induced obese C57BL mice	13 weeks on HFD	↓AG, GHSR1a↓	[89]

*Note:* AG secretion is increased (↑) and decreased (↓).

Abbreviations: AG, acyl ghrelin; CSDS, chronic social defeated stress; CUMS, chronic unpredictable mild stress; EPM, elevated plus maze; FST, forced swim test; GHSR1a, growth hormone secretagogue receptor 1a; HFD, high-fat diet; SD, Sprague-Dawley; SPD, Sprague-Dawley; TG, total ghrelin; WAS, water-avoidance stress; WKY, Wistar Kyoto.

## Data Availability

The data availability is not applicable to this review article.
